# More than “Just Breastfeeding”: a qualitative study on Indian mothers’ experiences of maternal autonomy and stress in infant feeding decisions

**DOI:** 10.3389/fgwh.2026.1799198

**Published:** 2026-06-04

**Authors:** Keerthana Balamurugan, Vijayalakshmi S

**Affiliations:** Department of English, School of Social Sciences and Languages, Vellore Institute of Technology, Chennai, India

**Keywords:** external criticism, maternal autonomy, postpartum mental health, realistic feeding experiences, structural and social barriers

## Abstract

**Introduction:**

Women's autonomy in infant feeding decisions is a fundamental element in maternal mental health, which is often disrupted by external factors like societal expectations, cultural norms, and institutional policies. The informed decisions made by the mothers in feeding their infants are often not made in a private environment, and they are not refrained from judgments and coercion.

**Methodology:**

This qualitative study analysed 11 mothers’ narratives of their infant feeding experiences through semi-structured, in-depth interviews. The participants gave individual informed consent for the interview, and the sessions were recorded with the participants’ consent. The collected narratives were summarised using thematic analysis, and the recorded interviews were transcribed into English.

**Findings:**

The thematic analysis revealed key themes like (1) Pregnancy Expectation vs. Postpartum Reality, (2) External Criticism from Family and Peers, (3) Emotional and Physiological Burden of Breastfeeding, and (4) Structural Barriers and Workplace Expectations.

**Discussion:**

The findings of the study highlight the interplay of external factors that influence the mother's feeding practices and decisions, which exacerbate maternal mental health. The findings reveal a crucial contrast between maternal reality and expectations, which are shaped by cultural beliefs and societal norms. The study supports the need to address these realistic challenges, which demand a supportive community, empathetic healthcare practices and structural reforms that will uplift women's autonomy and maternal mental health.

## 1 Introduction

Motherhood, even though it is culturally honoured and often viewed as a phase of unconditional joy and celebration, the underlying practical obstacles faced by new mothers are frequently overlooked. The recent romanticisation of motherhood has set unrealistic expectations for mothers, and these expectations frequently obscure the maternal realities (1–3). These expectations include being in perfect shape post-delivery, showering only love and care, and they do not include showcasing sleepless nights and constant breastfeeding, being tired and drained. All these expectations portray motherhood as effortless and undemanding, with mothers on social media often highlighting only the pleasant aspects while downplaying potential challenges. In reality, mothers are often exhausted because of the poor sleep cycle and constant breastfeeding, which often does not make it to social media. This leads to a perception that motherhood involves a dual life, with a constant switch between socially approved characteristics and the true attributes of motherhood (4, 5).

Mothers who go through significant changes during the postpartum period find these expectations placed on them to be overwhelming and unattainable. Among the major expectations placed on motherhood, decision-making regarding breastfeeding is a potential influencing factor in maternal distress. While breastfeeding is a highly personal and intimate choice, the breastfeeding mother often does not have the autonomy to make this decision, as various cultural and social pressures frequently influence this decision (6, 7). One of the major factors influencing this decision worldwide is different health authorities, as they suggest breastfeeding provides multiple nutritional and health benefits for both mother and baby. According to the World Health Organisation (WHO), breastfeeding is described as the ideal pathway to a child's nutrition and immunity, and it has also recommended that the optimal period for exclusive breastfeeding is six months (8). The United Nations Children's Fund (UNICEF), in collaboration with the World Health Organisation (WHO), recommends that breastfeeding for infants should be initiated within 1 h of birth and continued exclusively for six months and after six months, it is suggested to consider breastmilk as the supplementary food. The primary reason for predominantly endorsing breastfeeding is that it offers numerous health benefits, including the prevention of viral infections, reduction in newborn mortality, decreased risk of malnutrition, and support for brain development (9, 10). These regulations, set by health authorities, are evidence-based and were not formed considering the practical and psychological challenges faced by new mothers. Breastfeeding is often assumed to be an effortless experience, which creates an assumption that women who breastfeed are free from cultural and social pressures, and their autonomy in decision-making is not questioned.

Along with the health authorities, cultural narratives surrounding motherhood have led women to believe that breastfeeding is the most preferred way to connect emotionally with the baby, and it has been considered the epitome of motherhood (11). Although global health authorities and traditional narratives endorse exclusive breastfeeding for the first six months, they do not address the realistic challenges that women endure when they cannot breastfeed naturally. Women who are unable to breastfeed often face practical difficulties, such as lower milk supply, latching issues, breast pain during feeding, later onset of milk glands, etc. (12–14). Since greater emphasis is placed on exclusive feeding, women who fail to breastfeed naturally often feel guilty and anxious about not meeting the ideal qualities of a “perfect mother” (15, 16). These women usually internalise the socially approved traits of a perfect mother, and when they cannot attain those traits, they are frequently met with feelings of regret, shame, and inadequacy (17). A recent study on the impact of stigma on first-time mothers' breastfeeding has projected how women are met with feelings of negativity and lower self-esteem when they cannot breastfeed their infant. The study has shed light on the key difference, where mothers who “chose not to breastfeed” were met with lower public stigma and mothers who were “not able to breastfeed” were met with higher rates of public and personal stigma (18). This also denotes society's ignorance towards formula feeding and failure to acknowledge mothers' mental health who are unable to breastfeed. Though formula feeding is prescribed as an alternative method by the health professionals, the family setting does not create a comfortable atmosphere for mothers to feed formulated milk.

In many families, decisions related to breastfeeding are often not made in a private environment, as socio-cultural factors such as family beliefs, cultural norms, and religious rituals contribute to the mother's choices (19). For instance, a qualitative study on sociocultural beliefs surrounding antenatal care pointed out various superstitious beliefs, such as avoiding hot food and sweets, thinking that would harm the baby, indulging in prayers for diversion, avoiding screen time and noise, prohibiting husbands from entering the labor ward, not allowed to expose their face to others, not allowed for public gatherings or breastfeeding in front of others (20). All these superstitious beliefs are prevalent in many communities, which impact the breastfeeding environment. When it comes to family beliefs, family members' interference plays a massive role in influencing the mother's breastfeeding decision. The family members typically pass on the generational dynamics of motherhood to the next generation, which new mothers are expected to follow, such as breastfeeding the baby for more than a year, as it is believed that babies need greater immunity to survive (21). However, the decision to feed breastmilk or formula, or to wean off breastmilk, is entirely up to the mother. These familial settings are unquestionable and are deeply rooted in familial authority. Additionally, economic factors—especially in low-income countries where health information and resources are limited—and the lack of private space for nursing in workplaces and inadequate maternity leave also impact the breastfeeding decision (22). Amidst these limitations, when a mother makes a private decision on feeding her infant, it is often questioned or judged because of the deeply rooted belief that breastfeeding, irrespective of its emotional and physical toll, is being practised as a moral expectation across generations. The dominant healthcare and cultural discourses surrounding breastfeeding have given little room for considering women's autonomy over their bodies, their emotional well-being, and freedom of choice during pregnancy and postpartum. With the existing literature, many studies are focusing on the quantitative data on exclusive breastfeeding barriers, but there are only a few qualitative studies that exclusively focus on the relationship between women's autonomy in breastfeeding and barriers to exclusive breastfeeding. Therefore, the proposed article aims to provide an evidence-based study using mother-centred experiences to identify the relationship between the barriers to breastfeeding and the psychological distress it creates among breastfeeding mothers. The article highlights how these hindrances limit women's autonomy over their bodies, and it further decodes the perceptual shift on breastfeeding, on how a personal decision can be changed depending on structural, cultural and social barriers.

## 2 Materials and methods

### 2.1 Study design and setting

The study employed an exploratory qualitative design, which is well-suited to interpreting complex interpersonal and psychological realities that are already prevalent but not yet extensively researched or documented. The research design aimed to deepen understanding of the multidimensional aspects of breastfeeding barriers, including psychological, physical, social, and cultural factors, which are complex and often under-researched (7, 23–24). The study was conducted at the participants' workplace setting in Chennai to determine how breastfeeding mothers efficiently juggle between workplace responsibilities and nursing routines, which also provided direct access to comprehend the complex issues faced by breastfeeding mothers. The setting allowed the researcher to interpret a pragmatic context where practical challenges like inadequate maternity leave, limited breastfeeding facilities, and professional expectations play a significant role in influencing the mother's breastfeeding decision.

### 2.2 Study participants and sampling

Participants for this study were selected using a purposive sampling strategy, in which breastfeeding mothers who met the inclusion criteria were identified through personal face-to-face interactions. The inclusion criteria include participants who were exclusively or partially breastfeeding and mothers who were going through their postpartum period, and other characteristics are mentioned in Table 1. The finalised participants were willing to participate in individual interviews, which yielded in-depth, realistic accounts of their experiences with breastfeeding. The overall set of participants consisted of women with diverse feeding experiences, which directly met the prime objective of the study—to understand the relationship between barriers to breastfeeding and the psychological distress those barriers generate.

**Table 1:** Participant characteristics.

Participant name	Age	Education	Employment status	Postpartum time	Feeding method
Participant 1	29	Post Graduate	Maternity leave	1.5 yrs	Breastmilk
Participant 2	35	Doctorate	Working	1 year	Formula Feeding & Breastmilk
Participant 3	27	Post Graduate	Working	8 months	Formula Feeding & Breastmilk
Participant 4	30	Doctorate	Working	10 months	Breastmilk
Participant 5	32	Doctorate	Working	5 months	Breastmilk
Participant 6	28	Under Graduate	Maternity leave	11 months	Breastmilk
Participant 7	29	Under Graduate	Maternity leave	6 months	Formula Feeding & Breastmilk
Participant 8	33	Post Graduate	Working	1 year	Breastmilk
Participant 9	26	Under Graduate	Maternity Leave	9 months	Formula Feeding
Participant 10	35	Doctorate	Working	9 Months	Breastmilk
Participant 11	29	Post Graduate	Maternity Leave	5 Months	Breastmilk

### 2.3 Data collection

Semi-structured and In-depth interviews were conducted by the researcher to explore the sensitive and complicated experiences of breastfeeding. The feeding mothers were assured a safe and free space to discuss their personal experiences about nursing. The duration of each interview lasted for around 45–50 min, with open-ended questions, and it was conducted in the participants' preferred language, either English or Tamil. With informed consent, all interviews were audio recorded and transcribed. The open-ended questions were prepared in accordance with the prevailing literature and the study's goal, which interrelates societal & workplace pressures, cultural expectations, and the practical and emotional challenges faced by breastfeeding mothers. A total of 15 participants were selected for the study, of whom 11 were willing to participate, and their names were kept anonymous at their discretion.

### 2.4 Data analysis

The interviews, which were conducted in Tamil, were transcribed by the researcher. Braun and Clarke's thematic analysis was selected for the study. The interview transcripts were analysed through the following six steps,

1. Familiarising the collected data
  The transcripts were repeatedly read by the researcher to recognise recurring patterns in their experiences, such as lack of sleep, psychological distress, familial discrimination etc.
2. Generating initial codes
  After identifying the common patterns across the transcripts, the selected patterns exhibited significant phrases such as “night feeding routine”, “maternity leave” and “judgemental in-laws”. All these codes were taken into consideration for generating broader themes.
3. Theme Search
  In this step, the selected codes were segregated, and broader themes were formed based on that. For example, codes like “maternity leave” come under the main theme of structural barriers and codes like “judgmental in-laws” come under the theme of external criticism**.**
4. Reviewing themes
  The quotes generated from the transcripts were reviewed and segregated under the suitable theme. The data was reviewed thoroughly to check whether the selected quotes are connected with the theme and contain a clear distinction between them. For example, before the code “the night feeding routine” was inserted into the theme of “pregnancy expectations vs. reality”, the specific participant's quotes were reviewed for the connection between the code and the theme for accuracy.
5. Defining themes
  In this step, every key theme was assigned a meaning and a role of exhibiting certain characteristics. For example, when considering the theme of “External criticism”, any emotion related to the psychological distress caused by the unsolicited opinions given by the peers or in-laws can be framed under this theme. So, this theme projects characteristics such as mental distress and unspoken emotions of feeding mothers, which are caused by external factors.
6. Final report
  After finalising the gathered data, the final structured report will be discussed in both the results and discussions sections with the participants’ relevant excerpts to each corresponding theme.

### 2.5 Ethical approval

To increase the credibility of the study, the generated data and the derived themes were given special attention. The study maintains confidentiality throughout the study by ensuring anonymity, and all the personal details related to the participants were removed, and each participant is named by a number. This study was conducted in accordance with the local legislation and ethical research principles and ensures participant confidentiality. The study has obtained ethical approval from the Institutional Ethical Committee for Studies on Human Subjects (IECH), and written informed consent was obtained from all the participants before the data collection. Participation in this study is entirely voluntary, and the researcher has ensured to follow all the institutional ethical guidelines.

## 3 Results

Based on the analysis, the study arrived at four key themes, which are discussed in detail below.

### 3.1 Theme 1: pregnancy expectation vs. postpartum reality

Most of the mothers felt a significant gap between their pregnancy expectations for lactation and their postpartum reality. Though women are prepared for breastfeeding during their pregnancy, practical challenges like suckling, difficulty in latching, and inverted nipples are sensitive issues that are out of their control.

“Even though I attended webinars and lectures on breastfeeding positions and procedures, I could not do anything with what I learnt because of my C-section delivery. My baby did not know how to suckle, and I could not feed her in the position I had in mind. Nothing really worked in reality”. (Participant 1)

“My mother taught me how to feed the baby before my delivery, but once he was born, even with her instructions, I was not able to feed him properly because when it comes to breastfeeding, there are no practical classes—only theory classes” (Participant 4)

Few mothers were found with feelings of guilt and frustration of not being able to feed their babies at the time of birth, as their pregnancy beliefs were influenced by the idea that breastfeeding was the only preferred option to connect emotionally with the baby:

“Though my milk glands started secreting at the seventh month of my pregnancy, the initiation of breastmilk took time, and I was forced to feed her formula milk”. (Participant 9)

“I had this beautiful vision of feeding my baby until the age of 2, just like how they do in the movies, advertisements, and social media. But after he arrived, I came to know my breastmilk was thick, and I had to do combined feeding, which felt exhaustive”. (Participant 5)

Mothers, particularly during the exclusive breastfeeding phase, found it overwhelming to cope with their sleep cycle as babies have to be fed every 2 h, including nights. Although they are generally instructed during their pregnancy about this, when it comes to reality, it interrupts their sleep cycle, and mothers often feel irritated and detached from the baby:

“She used to cry all night, and I had no energy to feed her during the night. Even though I was at my mother's place, whenever she cries, I feel like I don’t want to hear that sound. My sleep was completely disturbed, and I stayed grumpy the whole day”. (Participant 8)

Night feeding is one of the significant phases during postpartum that highlights decreased sleep, depressive mood, and a feeling of being drained physically. These emotions can be balanced if the mother is provided with enough support from her family and partner. Most of the participants in the study were left all alone during the nights to face the reality:

“I did not receive any support from either of our families. I fed her all alone during the nights, crying and seeking help. But nobody was around, and I felt helpless”. (Participant 1)

“I was just waiting for her first birthday so that I could get rid of this night feeding. At times, even after feeding, she would refuse to sleep, and it would take hours to soothe her, which made me feel constantly tired”. (Participant 10)

“I did not even know postpartum depression could be a psychological condition. I was exhausted, feeding him monotonously, and I always questioned myself — Is this the life I wanted for myself? Nobody warned me about this tiring journey”. (Participant 2)

A few participants also reported how their postpartum reality changed when the baby was born underweight and was kept in the NICU for recovery. During this time, mothers had to pump the breastmilk on demand and also had to breastfeed, which placed both psychological and physical distress:

“Whenever I saw him at the NICU, my heart skipped a beat, and I did not even imagine I would pump my milk and feed him. He was a healthy baby in my tummy, and I thought my breastmilk would be enough for him”. (Participant 4)

“She was losing weight each day, which scared me more, and the NICU visits for feeding were the only time I was looking forward. On demand, she was fed with both breastmilk and formula milk to gain weight, which was the opposite of what I had planned during my pregnancy”. (Participant 1)

### 3.2 Theme 2: external criticism from family and peers

The discussions on external criticism faced by the mothers revealed that it had an enormous effect on their feeding decisions. The uninvited opinions given by family members and friends often acted as a strong barrier for women to choose breastfeeding over formula feeding:

“When I resumed back to work after his 6th month, one of my colleagues commented, ‘Are you heartless and numb? How can you leave your 6-month-old at home and return to work? She was against combined feeding, which irritated me”. (Participant 2)

Mothers shared that their feeding decisions were constantly questioned, and their emotions were invalidated by their family members:

“Even though my supply was good enough, I was repeatedly criticised for a lower supply, and my family forced me to feed her formula milk. I tried my best to avoid it for the first six months, as her health was good, but post six months, when I had to resume work, they fed her formula milk as a supplement, which I could not take control of”. (Participant 3)

Participants shared that even after the doctor's advice, their decisions on feeding and food choices were often denounced:

“I was put on a strict diet by my family, as she was having colic and reflux issues, and I tried quitting meat intake. Whenever she cried or threw up, everyone started questioning my feeding and food intake choices, which was upsetting and agitating”. (Participant 6)

“I tried all sorts of feeding-friendly food to boost my supply, but my supply was not enough for her. So, I tried combined feeding, which received everybody's attention at my in-laws’ place, and I was constantly criticised for giving her ‘artificial milk’—that's what they call it” (Participant 7)

One of the remarkable issues that noticeably persists among families is the infant's weight. Generational beliefs, such as infants with underweight, are characterised as innately unhealthy and infants born with normal and higher body weight are considered healthy babies. This particular belief has impacted many mothers' maternal mental health and invites unsolicited opinions and concerns about the baby's feeding practices:

“No matter how much I feed her day and night, she just does not gain weight. The doctors told me she is healthy, but her appearance did not convey that. My family members criticised me for not feeding her enough, even after cluster feeding her. I could not just control my anger whenever the topic was about her weight and about my feeding routine”. (Participant 8)

Another mother added:

“She was so tiny when she was born, and my family expected her to be a chubby baby since I was an obese person. When she came out like a tiny person, even though she was beautiful to my eyes, my family members were really not happy with her weight. They kept questioning my eating habits, my feeding practices, and I could not even directly express my anger towards them” (Participant 9)

### 3.3 Theme 3: emotional and physiological burden of breastfeeding

Most of the participants explained breastfeeding as a tiresome process, and the whole process of breastfeeding was often associated with the feeling of agitation and maternal guilt:

“I was completely frustrated when it came to feeding him; he was addicted to breastfeeding. He will not stop, and I was cluster feeding him. He needs breastmilk every day, and he used to bite my nipples whenever he was cranky. Now, this was something I wasn’t prepared for” (Participant 11)

“I used to feed her during the nights with no support, even my partner would be sleeping. Every 1.5 h, I have to wake up and feed her, which was burdensome, and I felt helpless. I felt agitated whenever she cried for milk during the night. I went to the extent of hitting her when she was just 25 days old. I still have the mother's guilt for that” (Participant 3)

Mothers shared that the reality of breastfeeding was highly contrasting when compared with the societal expectations of breastfeeding:

“My supply was low, and she was crying for breastmilk. Within a few hours after her birth, she developed jaundice because of a lower milk supply. I felt highly guilty the moment she became sick, as this was not what I expected. I always thought I ate a lot of fish, so obviously my supply would be high, but everything went in vain”. (Participant 6)

“I just felt relieved when she was born. I was enjoying motherhood for 4 months since I had people to take care of me. But after that, I went into depression because of monotonously feeding her. I had no other routine other than feeding her and taking care of her. I even had suicidal thoughts because of this tiring routine with zero support” (Participant 7)

“I was always found grumpy because of feeding him endlessly. Even though my husband took care of him during the day, I have to take care of him during the night. I was just waiting for his sixth month, so that I could have some rest. I showed all my frustration and my anger only towards my cook and my mother, I know that's not right, but yeah, that happened” (Participant 5)

Mothers shared that they thought breastfeeding would be a pleasant experience, as shown in films and on social media. Even healthcare providers or family members don't generally share the reality of breastfeeding experiences:

“When I fed him for the first time, he pulled my nipple, and I pushed him away in pain. I never knew something like this would happen, as I was not aware that breastfeeding includes pain and patience. My grandmother did not warn me about this” (Participant 11)

Another mother added:

“She was born underweight, and I almost lost hope in myself. I was constantly feeling anxious as my supply was low, and the only way for her weight gain was my milk. I felt both anxious and pressurized to increase her weight, and whenever I could not meet that ideal weight gain, I felt like I was failing as a mother” (Participant 10)

### 3.4 Theme 4: structural barriers and workplace expectations

Most of the participants reported that an insufficient maternity break interrupted their feeding routine, which created an uncomfortable situation to feed formula milk:

“I never wanted to feed her formula milk; that is my personal opinion. Since my maternity break was only six months, my family fed her formula milk and did not allow me to store my milk. Now that kindled mom's guilt in me” (Participant 6)

Few reported a lack of privacy while feeding the baby:

“My workplace did not have a dedicated feeding room. Every time I fed him, I fed him only with clothes covered. I did not like people staring at him or me, even if that's a woman. I felt uncomfortable” (Participant 5)

Mothers shared that their work schedules did not align with the baby's feeding time:

“I used to feed him in the middle of my online meeting. I could neither handle him nor my team. I would balance both, and it was very embarrassing and exhaustive. They say motherhood can be managed effortlessly, all that is a lie in reality” (Participant 4)

“I worked a full day shift. But fortunately, I had breaks in between. So, I had to drive all the way back home to feed her and come back to my shift, which was draining. But I had no other option; I had to do it for my baby” (Participant 3)

“My entire routine changed because of her feeding routine. I had to take care of my family, baby and the job I had. Even though I resumed work after one year, I fed her whenever I found time in the day care. I could not get any rest, even though I had support; it felt like a humongous task for me” (Participant 10)

One of the participants added that she had to quit her job because of the rigid work schedules and lack of feeding-friendly rooms:

“I had to quit my job because there were no feeding rooms or day care to take care of my baby. My husband was working from the office, and there was nobody to take care of my baby at home. After my maternity break, I could not resume, and I quit my job because of this. They say motherhood is all about sacrifices, but deep inside you want a life for yourself too” (Participant 2)

Another participant shared the lack of knowledge on pumping the breastmilk, which drew unwanted attention at the workplace:

“When I fed him by pumping my milk, everyone at my workplace saw me differently. A few commented that I was weird because I could not feed my baby directly, and a few commented that I am going against natural ways of feeding. But nothing stopped me from feeding my child, though those comments were irritating me in the back of my mind” (Participant 7)

## 4 Discussion

The outcome of this study portrays breastfeeding as not just a physiological process, but it is often intersected with societal, emotional and cultural expectations, institutional and structural barriers. The findings indicate how these factors collide with the mother's maternal mental health and influence the feeding decisions. The study highlights how women's autonomy is constantly questioned and hindered throughout pregnancy and the postpartum phase.

In Theme 1, Pregnancy Expectation vs. Postpartum Reality, most of the participants' experiences revealed that mothers were unprepared for the challenging reality and the expectations they had on pregnancy and postpartum were deeply rooted in the cultural narratives, social media portrayals, and community beliefs. Their experiences revealed that there was a constant pressure to meet the idealised motherhood standards, where breastfeeding was characterised as the quintessence of motherhood. Generally, mothers are frequently reminded that breastfeeding is a natural process that does not involve greater effort (25, 26). Such idealisation often creates an unfavourable environment in which successful initiation or adequate breastmilk supply is considered a positive indicator of an infant's feeding journey. The main objective of this theme is to delineate the contrasting differences between postpartum reality and prenatal expectations. Though most of the mothers were prepared mentally for breastfeeding with the help of healthcare providers and antenatal classes, when it came to reality, many participants found it challenging and tiresome. Participants reported a later onset of milk, lower milk supply, and difficulty in latching, and a few of them reported that C-section delivery was a major hindrance in feeding their infants. Mothers who undergo C-section deliveries often endure physical constraints while feeding, as they cannot carry out certain positions, or the way they hold their infant might be hindered post-surgery (27–29). On the other hand, participants with infants who were underweight had to endure the emotional strain of NICU visits. Mothers who had higher expectations for breastfeeding their infants experienced additional psychological stress when they had to switch from exclusive breastfeeding to combined feeding constantly (30). Participants recalled that this particular experience made them feel like a failure because, as per the societal norm, a “good mother” is recognised only when there is successful initiation of breastfeeding (31). Overall, the theme explains how mothers go through psychological stress when their feeding decisions are altered due to unforeseen postpartum reality, which is completely out of their control. The profound gap between prenatal expectations and postpartum reality is highlighted in the theme, as breastfeeding is comprehended as a socially ingrained experience rather than a fluid and situation-dependent experience.

The second theme's outcomes highlight that external pressure from friends and family predominantly influenced the mother's feeding decisions, and the unsolicited opinions often exacerbated their maternal stress and autonomy over their bodies. Participants reported feeling scrutinised by their in-laws or elderly women, and that their beliefs or perceptions were passed on to new mothers (32). These deep-rooted cultural beliefs left mothers with minimal agency, which predominantly resulted in submissive acceptance despite having personal preferences in feeding their infants. Participants shared that these cultural or societal beliefs surrounding infant feeding are generally disguised as the “right norm” or “right guidance”, but they primarily play the role of judgment and scrutiny. The theme decodes how external validation is seen as a determining factor in breastfeeding decisions, which aligns with the existing literature, where women often internalise the external criticism and suffer from feelings of inadequacy as a mother (33, 34). Mothers also reported that their decisions were frequently questioned or not validated, leading to a sense of agency denial and lower self-confidence. The external members' opinions were based on their own experiences, and they often rely on the concept that “breastfeeding is best”, by rejecting the evidence-based recommendations and the mother's preference (35). Despite being advised by the health professionals, mothers were denied the freedom to choose according to their comfort. A few mothers stressed the fact that their emotions were overshadowed by the fact that monotonously breastfeeding regardless of their preferences. Another striking element in this theme was the discrimination against the infant's weight, which created frustration and tension among the new mothers. Regardless of the health professional's recommendations, mothers often feel their self-efficacy was probed by equating them with their breastmilk supply, which led to an increase in postpartum depression and anxiety (36). One of the noteworthy features in this theme is the language used by the external members, such as “numb” and “heartless” denote the intensifying judgments that peers or family members pass when they do not adhere to the socially accepted attributes of a mother. These statements were passed down to new mothers when they returned to work, which reflects the narrow-minded approach of dedicatedly playing the role of a mother by exclusively breastfeeding. Additionally, framing formula milk as “artificial” exemplifies the prevalent misconception that, despite health authorities' recommendations, formula milk is still not accepted as the safest option for infants (37). Overall, the theme illuminates that external scrutiny functions as a potential stress aggravator among new mothers as it intensifies maternal mental health. The theme's findings suggest community-inclusive antenatal education on infant feeding, which obstructs the deep-seated societal beliefs and promotes the prioritisation of mothers' psychological well-being.

The findings of the third theme highlight the perception gap in how breastfeeding is promoted as the safest option, leaving little room to address the real challenges of breastfeeding. Although breastfeeding is broadly endorsed as the natural way, participants in this theme reported that it was the most demanding task, increasing both psychological and physiological stress for new mothers. The social media and cultural narratives' portrayal of breastfeeding as an effortless and peaceful activity was contradicted by the participants' experiences. Most mothers found breastfeeding physically draining and mentally overwhelming, which resulted in feelings of frustration, anger and guilt (38, 39). One of the mothers shared that she started hitting her infant out of frustration as she found breastfeeding tiring and burdensome without support. The impulsive pattern of expressing anger towards the infant was seen as a common practice among new mothers. This suggests a core significant gap in understanding the process of breastfeeding, where it is labelled as the feminine job (40), but in reality, collective support from family is an essential part of the successful continuation of breastfeeding (41, 42). The emotional burden of breastfeeding was magnified by the sleep deprivation, where one of the mothers shared that she had to wake up every 1.5 h to feed her baby, which felt helpless and provoked maternal mood disturbances. Sleep deprivation among new mothers, especially while exclusively breastfeeding, was the most common element, and it often resulted in the amplification of postpartum mental health issues (43). The theme also describes how participants were prepared for their breastfeeding journey by relying on the professionals' opinions, cultural beliefs, and media portrayals and how their experiences were contrasted. Experiencing nipple biting and pinching, demanding milk whenever needed, and low milk supply, all these were realistic challenges that were not demonstrated or advised during pregnancy. The cultural ignorance of breastfeeding education has paved the way for minimal discussions on the realistic challenges of breastfeeding (44). Despite mothers sharing similar experiences, they are not often shared or expressed, as the societal stigma often restricts open discussions on sensitive issues like breastfeeding. Candid discussions on social media surrounding the challenges of breastfeeding and its impact on postpartum mental health are highly essential for creating a supportive community for women (45). Another noteworthy dimension in this theme was the maternal self-blame and guilt, which arose in circumstances that were unmanageable. A few mothers shared that they internalised feelings of guilt whenever they were held responsible for the infant's underweight, colic and reflux issues, or whenever the infant developed a disease. This depicts the social norm of setting breastfeeding as a mere qualification or attribute of a good mother, rather than considering it as an act of primary nourishment (46). Overall, the theme suggests that though breastfeeding is an essential gateway for primary nourishment, the psychological challenges attached to it are generally neglected, which urges the need for an inclusive education on postpartum mental health.

The findings from theme 4, showcases how institutional hindrances, such as inadequate maternity leave, lack of privacy for feeding, and rigid work schedules, altered mothers' feeding decisions. When one of the participants shared that she was forced to feed formula milk to her infant as a substitute, it depicts how situations can become unmanageable when juggling between profession and postpartum. Inadequate maternity breaks often result in the substitution of breastmilk, which creates feelings of failure and maternal guilt among breastfeeding mothers (47, 48). A few participants described the discomfort of feeding in public in workplace settings due to a lack of privacy in the feeding rooms. Most mothers experience public feeding as shameful and embarrassing, and they often receive unwanted attention and stares (49). The lack of private space for feeding not only implies the institution's indifference towards breastfeeding but also denotes the societal stigma of sexual objectification of breasts. A few participants reported that breastfeeding in public was highly challenging because avoiding the public eye and feeding the baby at the same time felt chaotic. Though the mother's primary focus lies on the baby's hunger, the discomfort of receiving everyone's attention disrupts the breastfeeding confidence (50). Another significant dimension of this theme includes the resignation from work by a few mothers because of insufficient maternity leave and a dedicated childcare facility. Participants who resigned from their work felt that even after being labelled as the “perfect mother” for sacrificing their profession for the infant, they could not feel fulfilled, as motherhood does not predominantly revolve around the babies. When mothers were left only with the option of choosing their infant over their careers, they often endured feelings of identity crisis, lower self-esteem and a loss of self (51, 52). The lack of maternal-friendly spaces in workplaces reinforces the need to implement dedicated maternal policies, which include hygienic feeding rooms, flexible maternity leave, and childcare facilities, thus creating a supportive community for breastfeeding women (53). The theme also projects the stigma surrounding pumped milk, which was considered an unnatural method. When mothers shared that they received judgments and criticism for pumping their breastmilk, it portrays the lack of knowledge surrounding the benefits of pumped milk. Despite being prescribed by the health professionals, pumping breastmilk is not considered the natural way of feeding the baby, which implies society's negligence towards the various methods of lactation (54). Cultural beliefs about breastfeeding have made it a legitimate method of lactation by rejecting all the diverse methods by framing them as unnatural or unusual. Mothers who choose to feed their baby by the pumping method often receive less support and unwanted comments on their feeding choices, which implies the society's lack of understanding of the need to maintain an adequate milk supply for the infant (55). Many mothers find pumping comfortable and consider it the safest option, as it prevents milk ducts from getting clogged. In a broader context, the theme suggests that breastfeeding is neither predominantly confined to the mother nor seen as a maternal attribute. The outcomes of the theme also highlight that the continuation of breastfeeding also depends on the implementation of maternal inclusive policies, which allow women to sustain their identities instead of choosing between motherhood and career.

## 5 Conclusion

The study has explored the multifactorial dimensions that obstruct mothers' exclusive breastfeeding and has significantly highlighted the factors that influence infant feeding decisions, thereby depicting the lack of autonomy among feeding mothers. The findings have underscored how the autonomy of feeding mothers is frequently questioned and how it relies on various external forces, even though breastfeeding is considered a maternal choice. Although breastfeeding is considered the superior method of lactation, the mood disturbances and physical strain endured by mothers during this process are often intangible or ignored. The findings of the study underscore the need for realistic antenatal education, with transparent discussions of postpartum realities. This minimises the substantial contrast between prenatal expectations and the postpartum realities most participants in the study experienced. Further, the study emphasises the collective responsibility of family and society to create a supportive and safe environment for feeding mothers, with no room for judgment, baseless cultural beliefs, and deep-seated stigma. By dismantling the stigma surrounding breastfeeding choices, the rates of exclusive breastfeeding can be increased, and the postpartum mood disorders can be balanced. Further, it adds the need for institutions to relax rigid maternity break policies and implement dedicated feeding and day-care facilities. Thus, the study highlights that rather than placing breastfeeding as a maternal achievement, treating it as a simple source of nourishment by detaching societal beliefs would help women gain their autonomy in their breastfeeding journey.

## Data Availability

The raw data supporting the conclusions of this article will be made available by the authors, without undue reservation.
